# Biointerface Fiber Technology from Electrospinning to Inflight Printing

**DOI:** 10.1021/acsami.3c10617

**Published:** 2023-12-18

**Authors:** Wenyu Wang, Stanley Gong Sheng Ka, Yifei Pan, Yaqi Sheng, Yan Yan Shery Huang

**Affiliations:** † Department of Engineering, University of Cambridge, Trumpington Street, CB2 1PZ Cambridge, United Kingdom; ‡ The Nanoscience Centre, University of Cambridge, 11 JJ Thomson Avenue, CB3 0FF Cambridge, United Kingdom

**Keywords:** fiber printing, bioelectronics, tissue engineering, biofabrication, wearable devices, additive manufacturing

## Abstract

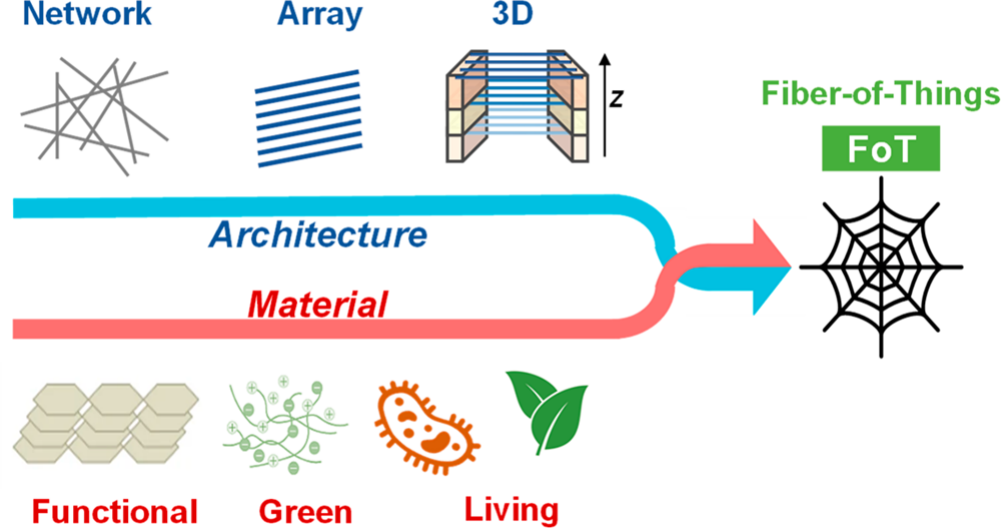

Building two-dimensional (2D) and three-dimensional (3D) micro- and nanofibril structures with designable patterns and functionalities will offer exciting prospects for numerous applications spanning from permeable bioelectronics to tissue engineering scaffolds. This Spotlight on Applications highlights recent technological advances in fiber printing and patterning with functional materials for biointerfacing applications. We first introduce the current state of development of micro- and nanofibers with applications in biology and medical wearables. We then describe our contributions in developing a series of fiber printing techniques that enable the patterning of functional fiber architectures in three dimensions. These fiber printing techniques expand the material library and device designs, which underpin technological capabilities from enabling fundamental studies in cell migration to customizable and ecofriendly fabrication of sensors. Finally, we provide an outlook on the strategic pathways for developing the next-generation bioelectronics and “Fiber-of-Things” (FoT) using nano/micro-fibers as architectural building blocks.

## 1 Introduction

From extracellular matrices (ECMs) in the connective tissues to silks spun by spiders and textile fibers weaved in fabrics, small diameter fibers widely exist in nature and have been closely associated with our daily life.^1−3^ These individual fibers have quasi-one-dimensional structures, with diameters smaller than ∼100 μm and length-to-width aspect ratios greater than ∼100, so that they could possess low bending stiffness and superior flexibility. Fiber-based and fibrous structures usually have favorable permeability and remodel-ability, making them well-suited for direct interfacing with biological systems.^4−6^ Therefore, numerous efforts have been devoted to creating fiber-based devices with functional materials, opening diverse applications from in vitro scaffolds to study cell and tissue mechanics^7,8^ to cell interfacing bioelectronics^9^ and on-skin and wearable sensors.^10−12^

In order for the fiber structures to be used as bioinstructive scaffolds or bioelectronic elements, individual fibers should be produced from functional materials with desirable chemical and sensing properties. The growth and maintenance of natural biological tissue rely on the dynamic support provided by the ECM, which could be regarded as a fiber-reinforced gel composite (the width of the fibers usually in the range of several micrometers).^13^ The fibril constituents of the ECM play a vital role in providing the biophysiological functionalities arising from the scale-dependent material chemistry, mechanics, and topography. Natural and biocompatible polymers, such as gelatin and collagen, could be used to produce ECM-environment mimicking fiber structures for studying basic biological processes, such as cell migrations,^14^ cancer developments,^15^ and neuronal regeneration.^16^ In addition, functional polymers with sensing capabilities have been explored to build bioelectronic and wearable sensors. For example, a range of functional polymers have been developed to produce fiber structures with conducting or energy conversion properties, including poly(3,4-ethylenedioxythiophene):polystyrenesulfonate (PEDOT:PSS), polyaniline (PANI), and poly(vinylidene fluoride) (PVDF).^9,17−19^ Alternatively, metallic nanoparticles and carbonaceous fillers could be mixed with polymers to produce composite fibers containing a functional phase.^20,21^

Conventional fiber production methods used in textile industries are not yet adaptable to produce ultrathin fibers made from functional materials. In the research and development space, a range of techniques have emerged to produce nonwoven fibrous scaffolds made of micro- and nanofibers. Among them, far-field electrospinning (FFES) is a widely used facile approach that relies on strong electrical fields to generate ultrathin fibers,^22^ and in situ electrospinning could directly deposit fiber mats on skins for wound dressing.^23^ In addition to electrical fields, small fibers could also be initiated and produced by air flow (i.e., blow spinning^12,24^), centrifuge forces (i.e., rotary jet spinning^25^), and mechanical stretching (i.e., touch spinning^26^). These techniques enable the cost-effective and scalable production of fibrous scaffolds for tissue engineering models and skin electronics. However, the above techniques intrinsically lack controllability over precise fiber deposition and placement; thus, the produced fibrous structures could only achieve at most global coarse fiber alignment (Table 1).

**Table 1:** Summary of Typical Fiber Spinning and Patterning Techniques for Tissue Engineering Scaffolds and Bioelectronics

technique		fiber patterning ability	fiber production mechanism	voltage (kV)	fiber resolution	exemplary applications
Far-field electrospinning^33^		None to coarse fiber alignment	Electrical field	∼10–15	100s nm to 1s μm	Bone regeneration scaffolds and on-skin electronics
In situ electrospinning^23^		None		∼10–15	100s nm to 1s μm	Fibrous mats for wound dressing
“Spray” spinning^12^		None	Air flow	0	100s nm to 1s μm	On-skin strain sensor
Rotary jet spinning^25^		Coarse fiber alignment	Centrifuge force	0	∼1 μm	Fibrous scaffolds for heart model
Touch spinning^26^		Coarse fiber alignment	Mechanical stretching	0	∼100s nm to 5 μm	Fibrous cell culture scaffolds
NFES (Near-field electrospinning) and variations	NFES^34^	Individual fiber patterning	Electrical field	∼1	∼50 to 5 μm	ECM-mimicking membranes
	Dynamic NFES^35^	Coarse fiber alignment	Dynamic electrical field	∼2.5	∼300 nm	Self-powered broadband acoustic sensor
LEP (Low-voltage electrospinning) and variations	LEP^36^	Individual fiber patterning	Mechanical stretching and electrical field	∼0.05–0.23	∼100 to 2.5 μm	Living material fibers, soft biological membranes
	3D-LEP^37^	Individual fiber patterning		∼0.1	∼3 μm	3D cell culture scaffolds
	Batch 3D-LEP^38^	Individual fiber patterning		∼0.1	∼2 to 4 μm	Batch 3D cell culture scaffolds
Inflight fiber printing^39^		Individual fiber patterning	Mechanical stretching	0	∼2 μm	3D optoelectronics, wearable sensor, and bioelectronics

There have been growing interests to improve the “pattern-ability” and deposition precision of thin fibers, because the precise control of fiber assembly underpins the functionalities of biointerface and bioelectronic devices.^27^ For example, high-resolution and well-defined fiber patterning could be important for the study of biomechanics and neuronal regeneration,^28^ and designable fiber patterning could be needed for sensor and circuity designs and connections.^29^ However, challenges remain in fabricating fibers with desirable and tunable functional performance and then to orderly assemble them into arrays or three-dimensional (3D) structures in a scalable and controllable manner. Furthermore, with the increasing focus on sustainable technology innovations, the direct production and patterning of fibers from a solution phase emerges as a promising branch of biofabrication.^30^ This approach occurs under biologically compatible processing conditions, offering significant appeals because it eliminates the requirements of conventional energy-, time-, and waste-intensive fabrication processes typically associated with the nano- and microfabrication of quasi one-dimensional structures.^31^

**Figure 1:**
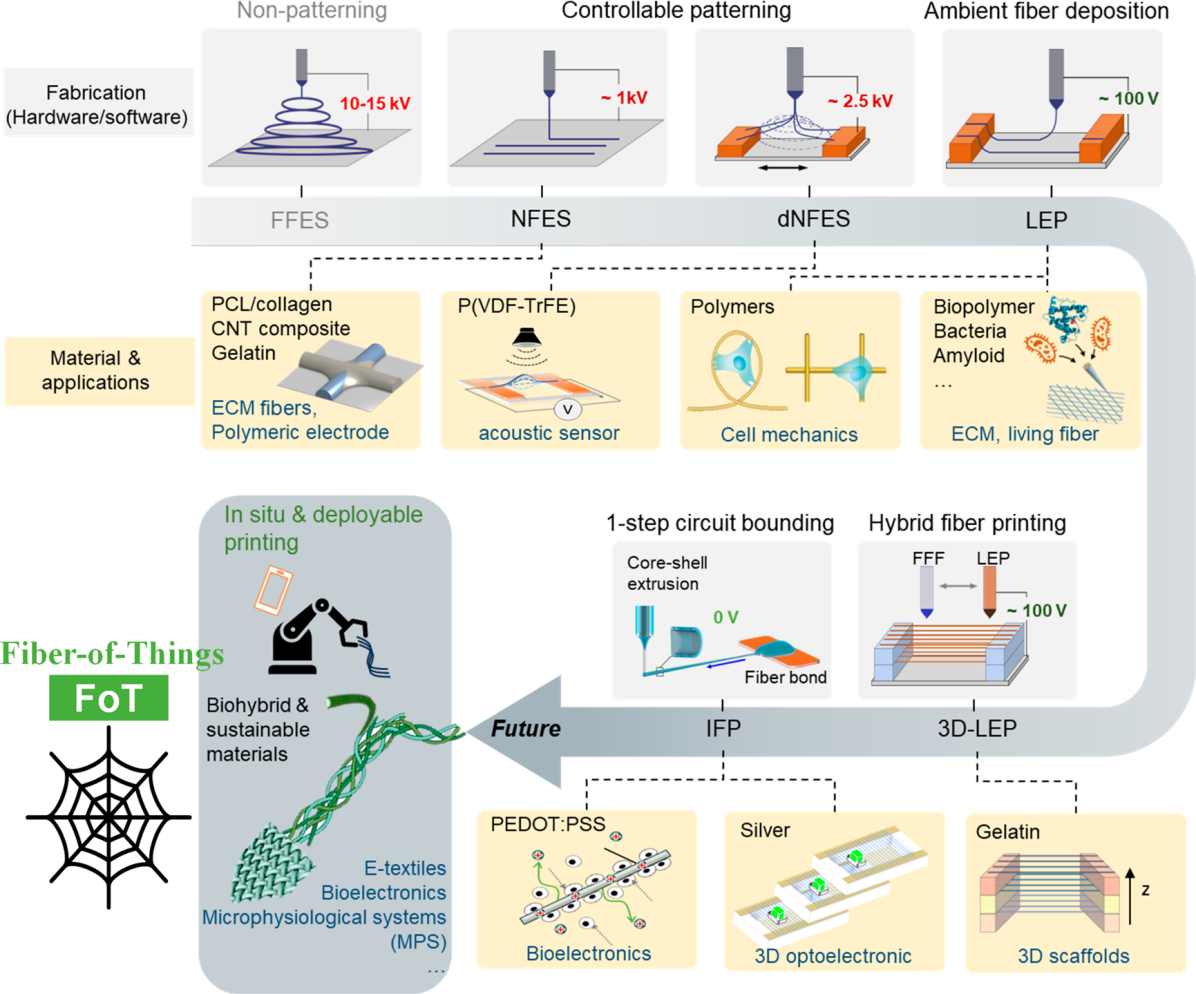
An overview of the fiber spinning and printing technologies developed by the authors’ research group.

The advancement of fiber patterning technologies evolves around two intertwining themes: the development of both “hardware” and “software” to enable versatile, scalable fiber architectural patterning, along with expanding the material library of “spinnable” solution formulations to create high-performance fibers. By shortening the tip-to-collector distance to eliminate the bending instability, near-field electrospinning (NFES) offers a controllable fiber deposition approach.^19,32^ However, the harsh processing conditions (i.e., static electric field ∼ MV/m, close to the dielectric strength of air) would limit the choice of functional materials suitable for this technique. Over the past decade, the authors’ research group has been developing high-resolution and designable fiber patterning techniques, evolving from low-voltage fiber patterning to integrating fiber printing with 3D printing and device/circuitry-level inflight fiber printing (Figure 1 and Table 1). Simultaneously, these fiber patterning approaches are demonstrated with a wide range of functional materials, including biopolymers, living materials, conducting and piezoelectric polymers, and metal–polymer composites. This Spotlight on Applications summarizes the development of a series of fiber patterning and printing techniques from our research group, while elaborating some of the biointerface scaffolding and sensing applications enabled by the fiber architectures. We will conclude by providing an outlook on the strategic pathways of upgrading the fiber material library and fabrication techniques for unlocking the future bioelectronics and electronic textile design and applications.

## 2 Designable Fiber Scaffolds by Electrohydrodynamic Writing

### 2.1 Near-Field Electrospinning and Its Variations

#### 2.1.1 Near-Field Electrospinning of Functional and Biomaterials

**Figure 2:**
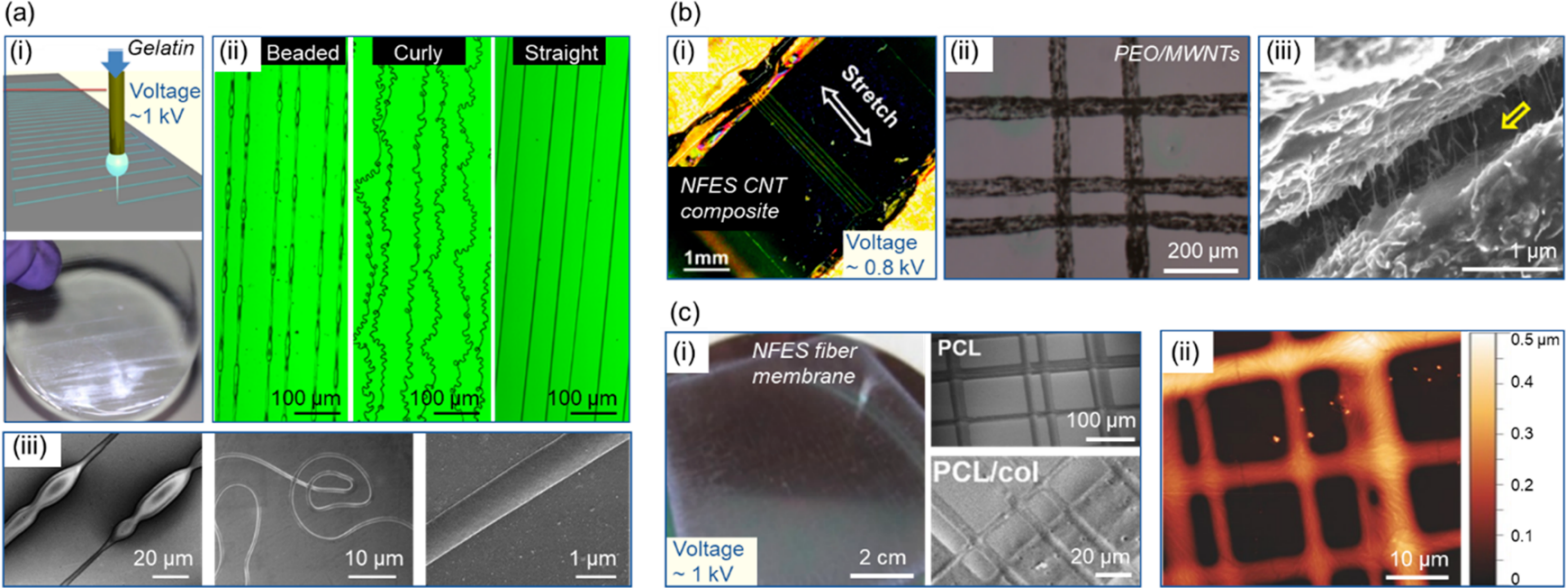
Near-field electrospinning of various biopolymers and carbon nanomaterials. (a) Versatile gelatin fibrous patterns created by the NEFS approach: (i) schematic illustration of the NFES direct-writing approach, (ii) beaded, curly, and straight gelatin fiber patterns, and (iii) scanning electron micrographs showing typical regions of the fiber patterns. Reprinted with permission from ref (40). Copyright 2014 PLoS One. (b) Multiwalled carbon nanotubes (MWCNTs) composite polymer electrode printed by NFES: (i, ii) microscopic images showing the composite fibers in suspension and on a substrate, (iii) MWCNTs bridging an open crack along the fiber axis (the MWCNTs are shown by a yellow arrow). Reprinted with permission from ref (42). Copyright 2012 IOP Publishing. (c) PCL/collagen interconnected fiber membrane fabricated by NFES: (i) photo of the thin film, (ii) SEM micrographs of the interconnected PCL and PCL/collagen fibers, and (iii) atomic force microscope characterization of the PCL fiber network. Reprinted with permission from ref (34). Copyright 2018 John Wiley and Sons.

By shortening the tip-to-collector distance to the range of ∼0.5 to 3 mm, NFES could realize fiber printing with a much lower voltage of ∼1 kV (Figure 2a-i); thus to enable controllable fiber deposition of various patterns.^40,41^ The chemical and rheological properties of the fiber solutions, such as conductivity, surface tension, boiling point, and viscosity, are crucial parameters that determine the fiber formation. Balancing these parameters and understanding their interplay are the keys to control the fiber morphologies in NFES. For example, as shown in Figure 2a-ii,-iii, beaded, curly, and straight gelatin fibers could be printed by tuning the fiber solution formulas (i.e., the solid concentration of gelatin and the composition of the solvents). In this study, it was shown that the solution conductivity (σ) is the most important parameter that would determine the fiber morphology from beaded (σ < ∼0.5 mS/cm) to straight (σ ∼ 0.75–1 mS/cm) and curly (σ > ∼1 mS/cm). The deposition precision of NFES could also be useful for producing fiber-based sensing and circuitry designs. As shown in Figure 2b, electrodes made of composite fibers of multiwalled carbon nanotubes (MWCNTs) in poly(ethylene oxide) (PEO) could be printed with precise control of the fiber placement and patterning configuration.^42^ Such NFES fabrication protocols provide a platform to direct-write polymeric electrodes and to integrate the fiber electrodes onto a variety of stretchable elastic substrates with high precision.

Notably, the short tip-to-collector distance of NFES (up to several millimeters) could lead to insufficient solvent evaporation, resulting in the fiber jet remaining solvent-rich when deposited. This feature could be utilized to create interconnected fiber junctions. As an example, orthogonal networks of polycaprolactone (PCL) fibers or PCL/collagen composite fibers were created using NFES (Figure 2c).^34^ This work presents a straightforward method to combine an ECM component with a biocompatible polymer into a network with robust junctions. Such networked fiber membranes could provide a collagen-rich cell culture interface for tissue engineering and potentially mimic the local topography and global confirmation of natural collagen fiber bundles (size in the micrometer-level range^43^) for in vitro culture. In addition, by tuning the fiber solvent properties (i.e., boiling point), the fiber surface textures could be controlled to mimic the nanotopographical surface morphology of the natural collagen fiber bundle.^44^

#### 2.1.2 Dynamic Near-Field Electrospinning

**Figure 3:**
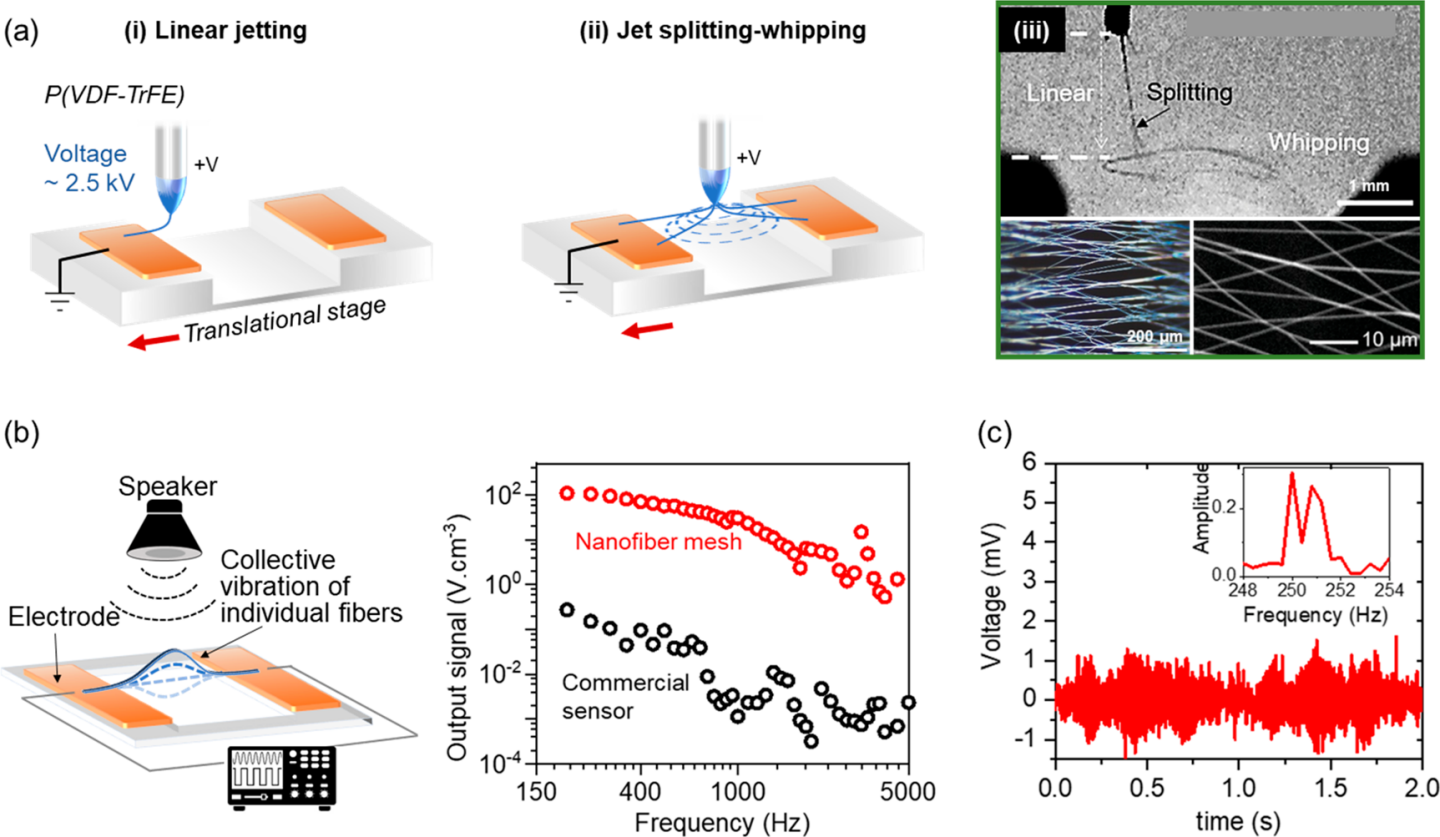
Process of dNFES and the piezoelectric nanofiber meshes as broadband acoustic sensors. (a) The process of dNFES is composed of (i) linear jetting and (ii) a transient process of jet splitting-whipping. (iii) A photo showing the jet splitting-whipping and microscopic images showing the suspended nanofiber meshs. (b) A schematic illustration of the sound sensing setup and the acoustic sensing bandwidth of the nanofiber mesh (equivalent film thickness ∼ 60 nm) compared with a commercial piezoelectric acoustic sensing dish (300 μm thick). (c) Signal output of the nanofiber mesh at 250 and 251 Hz at 70 dB and the FFT processed spectrum. Reprinted with permission from ref (35). Copyright 2020 John Wiley and Sons.

In the typical NFES setting, the unidirectional static field between the tip and collector restricts the fabrication of suspended fiber structures. By altering the placement of electrodes, our group developed dynamic near-field electrospinning (dNFES) to produce spanning and in situ poled piezoelectric nanofiber meshes made of poly(vinylidene fluoride-*co*-trifluoroethylene) (P(VDF-TrFE)) as broadband acoustic sensors.^35^ A typical feature of dNFES is that the fiber jetting status constantly varies between unidirectional deposition (resembling NFES status) and controllable jet whipping (resembling the FFES status). In the setup, the substrate, mounted on a translational stage, is composed of an electrode pair separated by an air gap. During fiber spinning, the stage moves in relative to the needle, so that the distribution of the static field is constantly changing (Figure 3a). When the needle is positioned above one of the grounded electrodes, the fiber jet is tethered onto the electrode substrate due to the unidirectional static field. Subsequently, when the needle is positioned above the air gap between the electrodes, the fiber jet splits and whips because the static field distributes evenly across the electrodes. Such transient jet splitting-whipping would result in a directional nanofiber mesh suspended over the air gap. Compared to conventional direct writing, dNFES offers the possibility to create low packing density and suspended nanofiber meshes (up to several millimeters of suspension distance). Inspired by the physical merits of spider silks as nonresonating and wide bandwidth acoustic sensors,^45^ the suspended piezoelectric nanofibers demonstrate self-powered and broadband acoustic sensing capabilities (from 100 to 5000 Hz with at least 1 Hz resolution) (Figure 3b,c). In the future, such broadband and permissive acoustic sensors could find potential applications in smart devices and the Internet of Things.

### 2.2 Low-Voltage Electrospinning for Building Biointerface Fiber Scaffolds

#### 2.2.1 Low-Voltage Electrospinning

**Figure 4:**
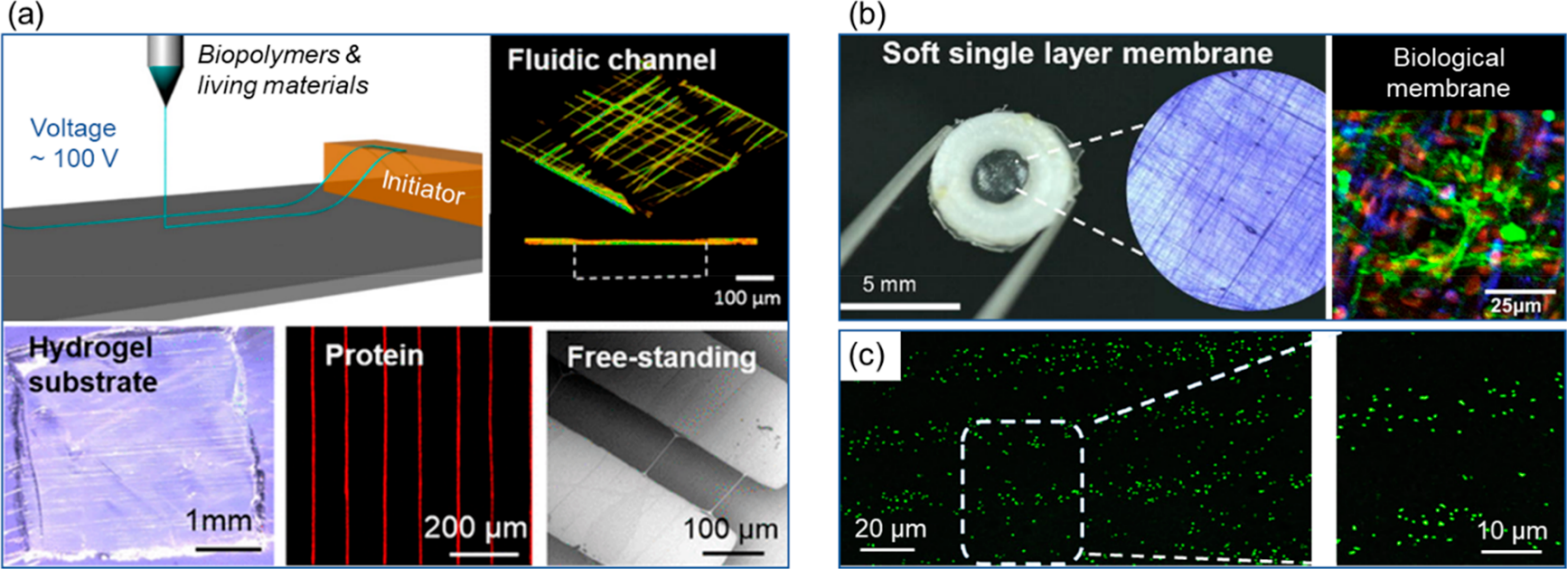
Low voltage electrospinning of biopolymers and living materials. (a) A schematic illustration of the LEP process and microscope images showing various fiber materials and patterns. Adapted and reprinted with permission from ref (36). Copyright 2016 American Chemical Society. (b) A suspended ECM-laden fiber membrane patterned on a 3D printed frame and immunofluorescence image of cell coculture of glomerular endothelial cells and podocytes on the membrane (red fluorescence: nuclei; green fluorescence: VE-Cad; blue fluorescence: podocytes). Adapted with permission from ref (46). Copyright 2018 Elsevier. (c) Confocal images of LEP patterned *E. coli* fiber arrays on glass. Reprinted with permission from ref (36). Copyright 2016 American Chemical Society.

The inherent high static field of NFES could induce damages to the functionalities of the fiber materials or the depositing substrates, especially for those delicate bio- and living materials. Continuous ultralow voltage electrospinning (LEP) offers an alternative route to create precise and designable fiber patterns with very low voltages (50–230 V) (Figure 4a).^36^ The initiator design in the LEP provides lateral mechanical stretching forces to induce the fiber jet from the solution droplets (Taylor cone); thus, the addition of low voltages is only required to maintain fiber patterning. LEP is compatible with a wide range of biopolymers and even living materials, such as polyethylene glycol (PEO)/water, polystyrene (PS)/dimethylformamide (DMF), polyvinylpyrrolidone (PVP)/ethanol, and bacteria laden solutions. In a study of LEP, a soft biologic membrane could be printed entirely made with ECM protein fibers (Figure 4b).^46^ In the dry fibers, urinary bladder derived decellularized ECM, consisting of up to 50% w/w collagen IV, laminin, and fibronectin, showed well preserved bifunctionality after the LEP process. Such ECM-mimicking fiber membranes could be useful for monolayer cell culture to generate functional tissue mimics such as endothelial layer and transmembrane coculture models involving glomerular cell types and endothelial cells. Advantageously, LEP enables ECM-rich composition in the fiber membrane, so that the Young’s Modulus could be tuned between ∼600 kPa and 50 MPa, enabling the potentials to tailor tissue-matching structural and mechanical properties for in vitro modeling and wound dressing.

The mild processing environments of LEP make it favorable for patterning biological elements such as living cells and bacteria. The lowered voltage and electrical field intensity make it possible to directly deposit the living material encapsulated fibers onto soft and biocompatible substrates, such as hydrogels. For example, living bacteria could be directly deposited on hydrogels or other microfluidic devices (Figure 4c). After fiber printing, the bacteria showed high viability and normal growth rate compared to reference groups.^36^ Although FFES approaches have been demonstrated for producing bacteria-laden fibers, there were mixed reports of the bacterial cell viability.^47−50^ In comparison, LEP offers a more stable and gentle processing route for living materials by minimizing the potential harm from the high voltage and electrical field.

#### 2.2.2 3D Low-Voltage Electrospinning

**Figure 5:**
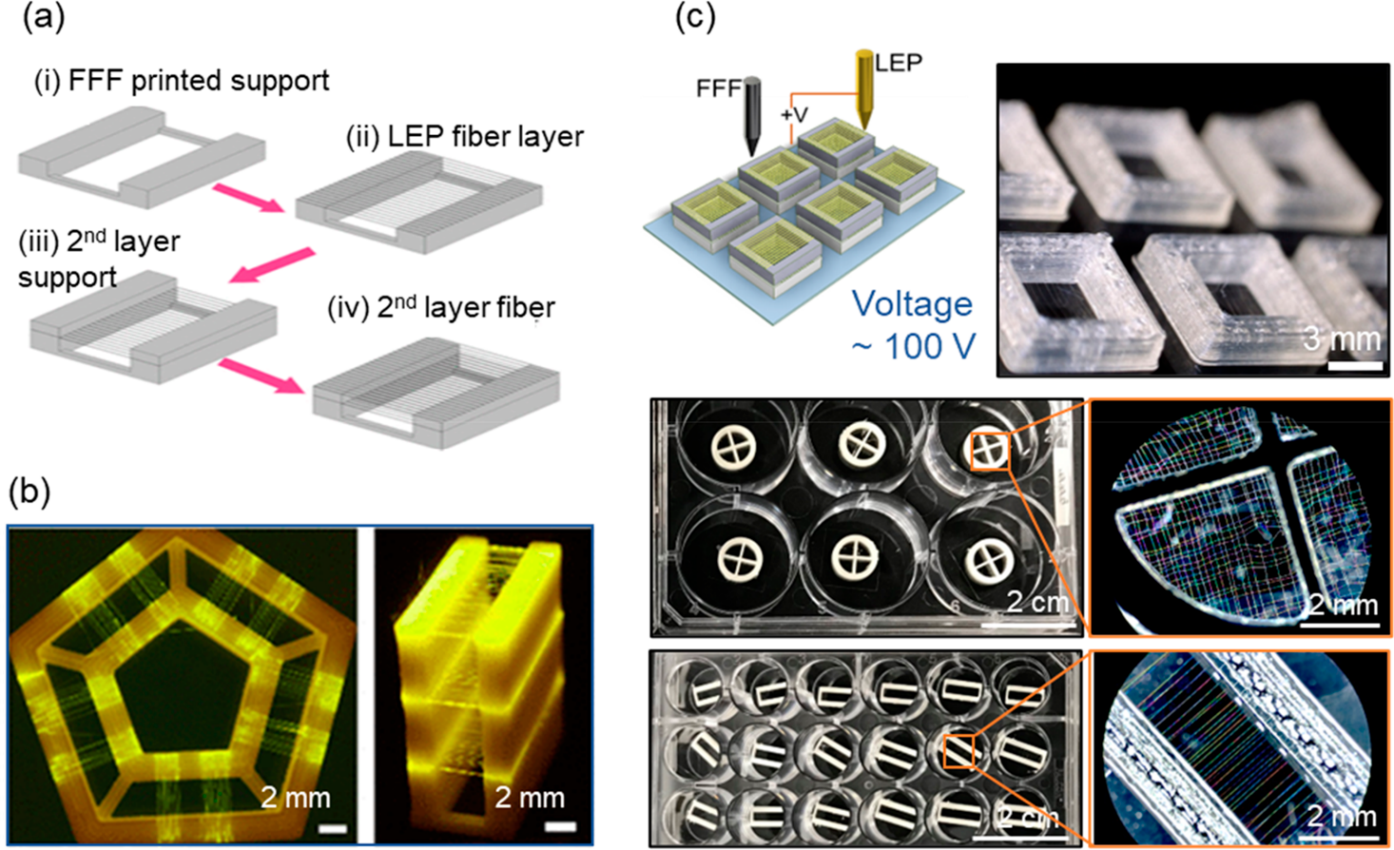
Fabrication of designable 3D fibrous structures. (a) 3D fibrous structures could be built layer by layer by altering the FFF and LEP processes. (b) Photos showing versatile 3D fibrous structures. Reprinted with permission from ref (37). Copyright 2019 American Chemical Society. (c) A schematic illustration of the batch 3D LEP process and photos showing the 3D fiber scaffolds being produced in batch manner with various designs. Reprinted with permission from ref (38). Copyright 2020 Elsevier.

Many of the existing fiber production methods specialize in producing planar fiber mats or films, while 3D fiber scaffolds hold prospects for a range of emerging fields, such as tissue engineering, sensing, and energy conversions. In order to address the challenge of producing 3D fiber scaffolds without hindering the design versatility and material choice, 3D low-voltage electrospinning (3D-LEP) was developed that integrates a fused filament fabrication (FFF) method with LEP (Figure 5a).^37^ 3D-LEP is capable of patterning vertically stacked layers of suspended mesofibers with designable structures and orientations (Figure 5b). The 3D-LEP technique unlocks the design capabilities of 3D fiber scaffolds. In addition to design versatility, scalability, repeatability, and device quality control are also essential considerations. We then demonstrate additive batch fiber patterning, as an efficient workflow to produce versatile 3D fiber scaffolds in a batch manner with minimized sample-to-sample variance (Figure 5c).^38^ This could especially be useful for in vitro study and bioelectronics because most biological experiments need to be performed in a batch manner with high standardizations. Customized fiber scaffolds could be conveniently designed and produced according to the requirements, for example, to be fitted with 6- to 24-culturing well plates. The configurations of the 3D-LEP and batch fabrication techniques have been made open to the public with technical design procedures.

## 3 Versatile Fiber Scaffolds for Cell and Tissue Mechanics Study

**Figure 6:**
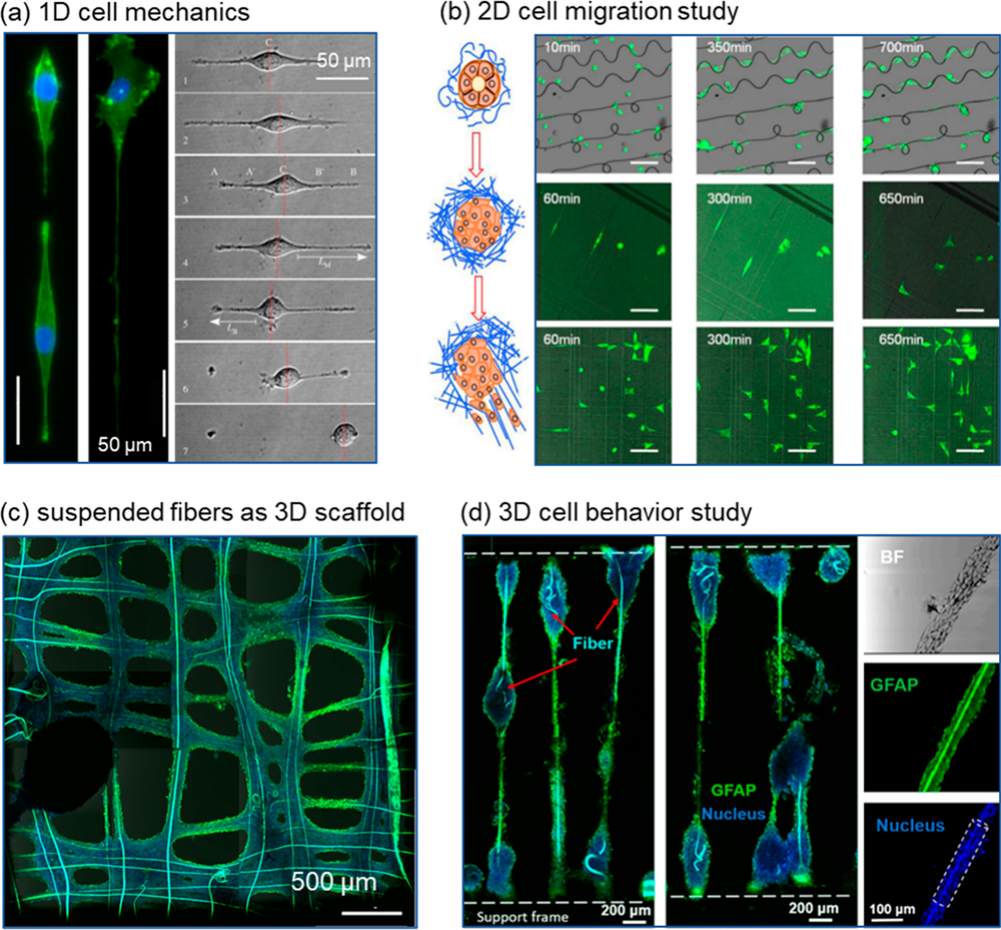
1D to 3D culture to study cell mechanics. (a) Time lapse images of the EA.hy926 endothelial cell migration process on 1D gelatin fiber (green fluorescence: F-actin; blue fluorescence: nuclei). Reprinted with permission from ref (51). Copyright 2014 The Royal Society. (b) Time lapse images of GFP-tagged MDA-MB-231 cancer cells migrating on 2D polystyrene fiber networks of various patterns. (c) Human glioblastoma cells U87 aggregated on gelatin fibers (green fluorescence: GFAP, a glial cytoskeletal marker; blue fluorescence: nuclei). Reprinted with permission from ref (37). Copyright 2019 American Chemical Society. (d) Immunofluorescence images of an ellipsoid-on-string formed by human glioblastoma U87 cells along the suspended gelatin microfibers (green fluorescence: GFAP, a glial cytoskeletal marker; blue fluorescence: nuclei). Reprinted with permission from ref (15). Copyright 2021 IOP Science.

With designable fiber patterns and materials, versatile biointerface scaffolds could be produced to better mimic the diverse microfiber environments in the native ECM, enabling cell and tissue mechanics studies from simplified one-dimensional to in vivo-like three-dimensional conditions (Figure 6). At the individual fiber level, the interaction between endothelial cells and the ECM fibrils could be recapitulated in vitro using gelatin fibers (Figure 6a).^51^ With various planar fiber patterns, the role of morphological cues provided by ECM could be used to study cell migrations. Cancer cell migration dynamics were observed using LEP produced fibers of different patterns, and it was found that the upper limit of the cell body minor axis determined cells’ fiber switching ability, revealing fiber displacement as a key factor to consider in scaffold design (Figure 6b).^52^ Suspended ECM protein-based fiber arrays were fabricated in situ on polymer frames, creating various patterns with a defined 3D structure. The biocompatibility of 3D ECM-based fiber array was demonstrated by culturing glioblastoma cells (Figure 6c).^37^ Furthermore, an aggressive form of brain cancer cell clusters was cultured on the scaffold, and rapid proliferation and guided migration were observed with highly aligned cell morphologies (Figure 6d).^15^ Those cell adhesive fiber arrays with controllable spatial displacement could lead to future applications in creating aligned and stackable cell arrays with multiple cell types for disease modeling and basic biological research.

## 4 Inflight Fiber Printing for Building Bioelectronics and Wearable Sensors

### 4.1 Infight Fiber Printing

**Figure 7:**
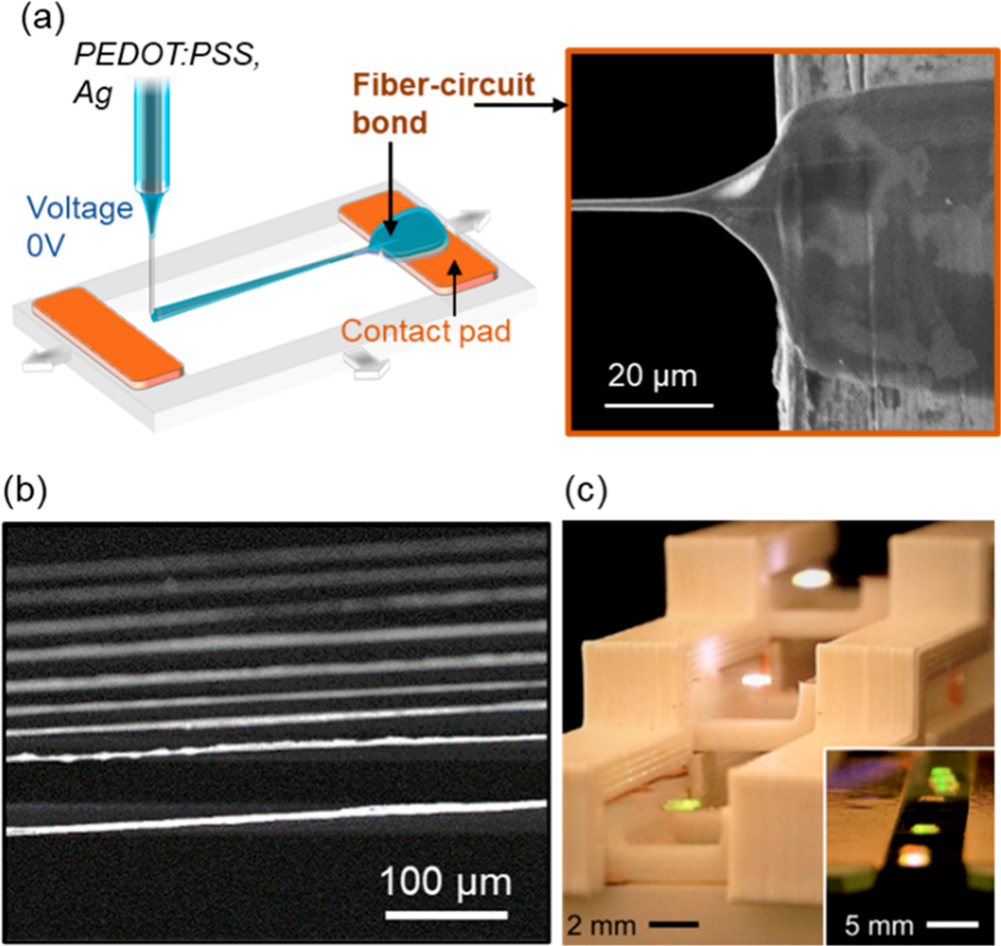
Inflight fiber printing for a one-step creation of the fiber circuit. (a) A schematic illustration of the iFP process, with an inset SEM micrograph showing the fiber bond region that connects the suspended fiber with the contact pad. (b) Typical suspended silver fiber arrays created by iFP. (c) Integrating iFP silver fibers with 3D printed plastic structures to create a substrate-free 3D circuit. Adapted and reprinted with permission from ref (39). Copyright 2020 AAAS.

In addition to structural scaffolding, fiber-based bioelectronics and wearable sensors could offer directionality, permeability, and cell attractiveness that are inaccessible from conventional rigid and thick film-based electronics. Orderly assembling fibers with embedded conduction and sensing capabilities could open the way for smart wearables, intelligent displays, and biointerfacing electronics.^53,54^ Various methods are available to produce fibers with conduction and sensing functions, such as chemically growing, electrospinning, wet spinning, and coating.^55−57^ However, these methods still struggle to achieve precise placement of high-performance fibers and fiber circuit connections that are essential for device-level integration. To address the challenges, we developed inflight fiber printing (iFP) as a one-step process to synthesis conducting ultrathin fibers (diameter ∼ 2 μm) with device-level circuit bonding.^39^ In the iFP strategy, core–shell fibers were synthesized where viscoelastic sizing polymer in the shell channel guides and encapsulates the delivery of conducting inks in the core channel (Figure 7a). Therefore, the composite fiber is formed with a highly pure conducting phase as the fiber core (PEDOT:PSS or silver micro/nanoparticles), enabling high conductivity without the need of postprocessing (the silver and PEDOT:PSS fibers could achieve conductivities of ∼10^6^ S/m and around 7000 S/m, respectively). Mechanical stretching force is the main driving factor for fiber formation in the iFP process, instead of static electrical force in electrospinning; thus, iFP could produce spanning and intrinsically substrate-free fibers of high spatial resolution (Figure 7b). The use of the sizing polymer and the core–shell extrusion design allows a variety of aqueous functional inks of low viscosity to be directly used for fiber printing without needing to tune their rheological properties with additives. In addition to conducting inks, natural biomaterials (i.e., protein solutions) could be used in their original solution status to form fibers.^58^

The solution drawing feature and the high precision fiber placement of iFP enable one-step fiber-circuit bonding without postprocessing. As seen in the inset SEM image of Figure 7a, a fiber bond is in situ formed during the iFP process, which serves as an electrical connection and mechanical anchoring for the freestanding fibers. The fiber bond on the circuit contact pad is much thicker (tens of micrometers in width) compared to the individual fibers, due to the wetting between the fiber solution and the contact pad. Such one-step processing exempts the need of postmanipulation, which could be difficult considering the small diameter of the individual fibers. The ambient processing conditions of iFP (<100 °C) widen the material selections for the substrates onto which the fibers could be directly printed and integrated. As an example, the iFP silver fibers could be printed to combine with a 3D printed plastic architecture (poly(lactic acid), melting point 150–160 °C) to produce substrate-free 3D circuits (Figure 7c).

### 4.2 Building Fiber-Based Biointerfacing Sensors

**Figure 8:**
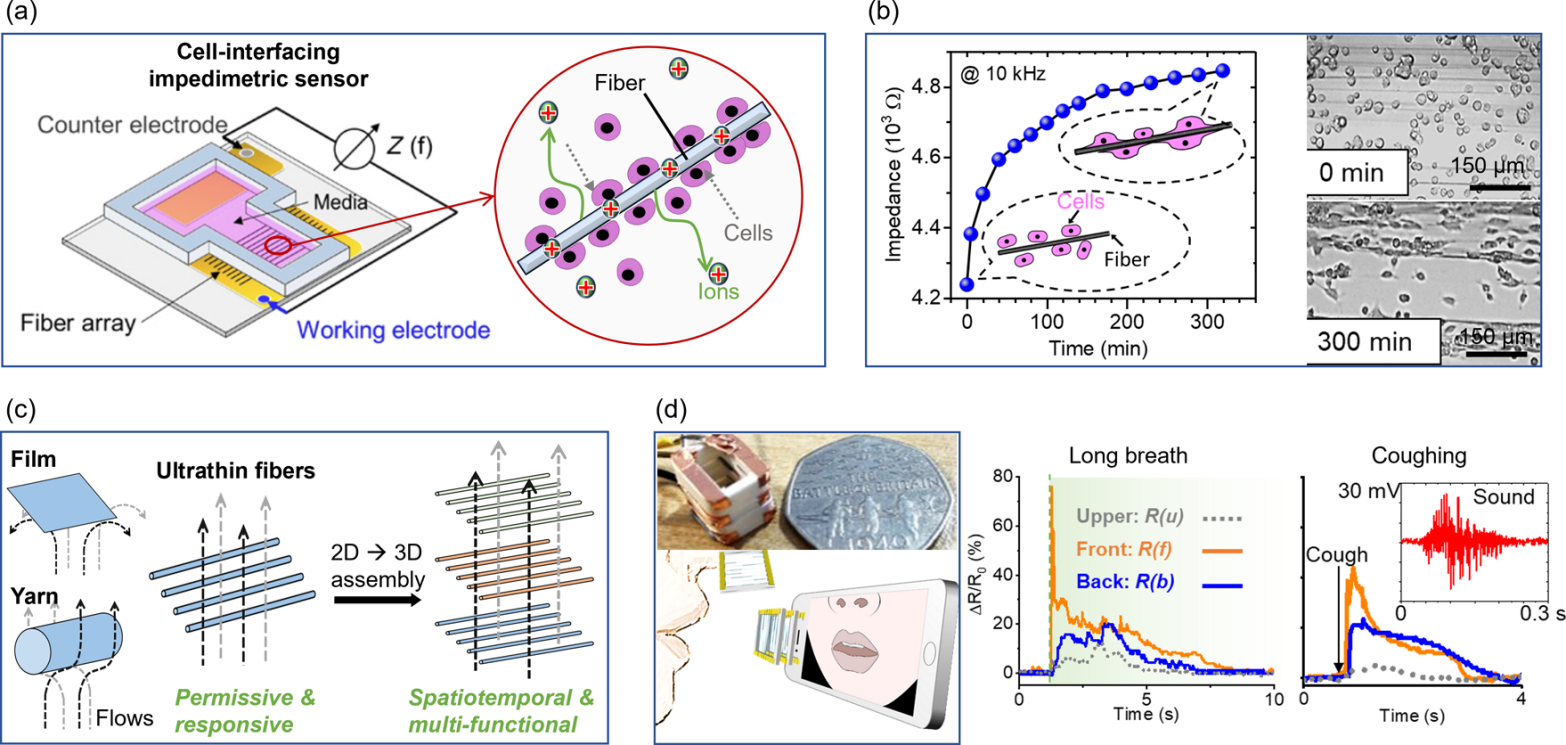
Fiber-based bioelectronic and wearable devices. (a) A schematic illustration of the iFP PEDOT:PSS fibers as a cell interfacing impedimetric sensor device. (b) The impedance of the cell-interfacing sensor increases when fibroblast cells attach and proliferate on the fibers. (c) A schematic illustration showing the substrate-free iFP fibers as permissive sensors to detect flows. (d) A fully printed spatio-temporal breath analyzer composed of multiple substrate-free sensing fiber layers that can detect breath strength and spatial distribution along with the sound of coughing. Adapted and reprinted with permission from ref (39). Copyright 2020 AAAS.

The permissiveness and flexibility of fiber-based structures make them particularly favorable for biointerfacing applications from in vitro cell and tissue interfacing bioelectronics to on-skin and wearable sensors. For example, electrospun nanofiber mashes, functionalized with metallic materials, have been used as sensors for monitoring dynamically pulsing cardiomyocytes in vitro^59^ and on-skin sensors for tactile pressure,^60^ biopotentials,^33^ and hand movements.^12^ Compared to conventional rigid or film-based sensing devices, these nanofiber meshes greatly minimize the mechanical restrictions or disturbances to the biological hosts.^61^ However, the electrospun nanofiber meshes are usually composed of randomly stacked fibers without alignment; thus, a stencil mask or microfabrication would normally be needed in order to form the sensing circuity with the fiber meshes. On the other hand, iFP fibers, with high resolution pattern-ability, could open a range of sensor architecture designs for biointerfacing applications. Thanks to the favorable conductivity and biocompatibility, the iFP PEDOT:PSS fibers could be used as cell interfacing sensors, which detect the cell presence and density on the fiber arrays through impedance spectroscopies (Figure 8a). The fiber array acts as both physical scaffolding to guide the cell attachment and alignment, and sensors to detect the cell dynamics on them (Figure 8b). This could pave the way for future studies in 3D cell culture with in situ sensing and stimulation. The ultrathin and substrate-free fibers created by iFP are permissive to flows, and the large surface area to volume ratio of the individual fiber permits fast sensing responsiveness. Therefore, the fiber arrays could be arranged to achieve spatial–temporal and multifunctional sensing (Figure 8c). As an example, a 3D printed wearable breath sensor made of PEDOT:PSS fibers and piezoelectric P(VDF-TrFE) fibers could detect human breath patterns, and also the sound of coughing (Figure 8d). Such lightweight, low-cost disposal devices could see promising adaptation in assisting mobile-health diagnostics and assessments.

## 5 Conclusion and Outlook

The rapid developments of material science and nanotechnologies expand the material library for producing various functional fibers, prospecting future biointerfacing fiber scaffolds to lay beyond structural support and sensing capabilities. From the materials’ perspective, the development of responsive materials, that undergo controlled and predictable changes in response to external stimuli, opens opportunities to fabricate scaffolds that could reform, repair, or regrow.^62,63^ Currently, extrusion-based printing is the main approach to fabricate 4D structures; thus, the structural resolution could be limited to hundreds of micrometers.^62^ In the future, integrating responsive or living materials with fiber printing techniques could realize 4D fiber biofabrication, which could be a promising strategy for producing biomimetic tissue engineering scaffolds and even artificial and biohybrid tissues. Transducer-type fibers (e.g., piezoelectric mechanical stimulation^64^ and light generation for optogenetic manipulation^65^) can introduce proactive stimulation into the system, adding additional input control for the next-generation fiber-based electronic devices. From the structure’s perspective, the device functionalities also rely on the fiber morphology and structural design to bridge between nano- and micrometer scale to the millimeter and centimeter scale. As described by this Spotlight on Applications, various fiber patterning methods have been developed for building designable fiber scaffolds from arrays to 3D formats. These techniques could further enable pixel/voxel-based bioelectronic “matrix” system configuration with high spatial resolutions to interact with tissues and cells.

On the whole-body scale, fabricating fibers and yarns with functional materials and their assembly as networks and devices could advance the future of e-textiles toward “Fiber-of-Things” (FoT).^66^ This hierarchical integration could enable multiplexed sensing and energy conversion, allowing for self-sufficient collection and analysis of information across multiple signal dimensions.^31^ For instance, a self-powered smart textile integrated with multifunctional fibers could simultaneously monitor multidimensional health situations, providing a comprehensive view of an individual’s physiological state in real-time. The ordered assembly and sensing textile designs would be especially important for decoupling the multidimensional signals.^67^

Driven by the need of customizable device design and the requirement of sustainability, future fiber-based biofabrication would develop toward on demand production while minimizing the environmental footprints.^68^ Artificial intelligence could provide efficient device structural and functionality designs to maximize higher customization freedom and supply chain robustness.^69,70^ Material- and power-light fiber printing technologies promise on demand and on site/in situ fiber fabrication (i.e., with a movable robotic printing platform commended by smartphones). Looking ahead, we can expect the emergence of multifunctional, environmentally friendly, and on demand fiber devices to revolutionize bioelectronic technologies for fundamental neuroscience research, 3D cell culture and microphysiological systems, and wearable sensors and “Fiber-of-Things” (FoT).
